# The Gut Microbiome–Estrogen Axis in Menopause: Implications for Body Composition, Cardiometabolic Health and Nutrition

**DOI:** 10.3390/nu18203330

**Published:** 2026-10-11

**Authors:** Oana Pătru, Silvia Luca, Andrei Păunescu, Bogdan Enache, Cristina Văcărescu, Dragoș Cozma, Simina Crișan, Andreea Bena

**Affiliations:** 1 Cardiology Department, “Victor Babes” University of Medicine and Pharmacy, 2 Eftimie Murgu Sq., 300041 Timisoara, Romania; oana.patru@umft.ro (O.P.); bogdan.enache@umft.ro (B.E.); cristina.vacarescu@umft.ro (C.V.); dragos.cozma@umft.ro (D.C.); simina.crisan@umft.ro (S.C.); 2 Research Center of the Institute of Cardiovascular Diseases Timisoara, 13A Gheorghe Adam Street, 300310 Timisoara, Romania; 3 Doctoral School, “Victor Babes” University of Medicine and Pharmacy, 2 Eftimie Murgu Sq., 300041 Timisoara, Romania; 4 Institute of Cardiovascular Diseases Timisoara, 13A Gheorghe Adam Street, 300310 Timisoara, Romania; 5 Department of Urology, “Pius Brînzeu” Emergency Clinical County University Hospital, 300723 Timisoara, Romania; ai.paunescu@gmail.com; 6 Discipline of Endocrinology, Second Department of Internal Medicine, “Victor Babes” University of Medicine and Pharmacy, 2 Eftimie Murgu Sq., 300041 Timisoara, Romania; bena.andreea@umft.ro; 7 Center for Molecular Research in Nephrology and Vascular Disease, “Victor Babes” University of Medicine and Pharmacy, 2 Eftimie Murgu Sq., 300041 Timisoara, Romania

**Keywords:** menopause, postmenopausal women, gut microbiome, estrobolome, estrogen metabolism, body composition, cardiometabolic health, precision nutrition, women’s health

## Abstract

**Background/Objectives**: Menopause is associated with profound endocrine, metabolic, immune, and microbial changes that may contribute to accelerated biological aging and increased cardiometabolic risk. The gut microbiome represents a potential interface between estrogen biology, host metabolism, inflammation, and systemic organ function. This narrative review examines the role of menopause-associated gut microbiome remodeling in microbiome-mediated metabolic aging, focusing on the estrobolome, microbial metabolites, organ crosstalk, and microbiome-targeted interventions. **Methods**: Relevant literature was identified through searches of PubMed/MEDLINE, Scopus, and Web of Science, supplemented by reference-list screening. Human observational and clinical studies, mechanistic investigations, systematic reviews, meta-analyses, and relevant guidelines were critically evaluated and synthesized narratively. No formal meta-analysis was performed. **Results**: Menopause-associated changes in gut microbial composition and function may influence estrogen metabolism through the estrobolome and modify the production of short-chain fatty acids, lipopolysaccharides, trimethylamine N-oxide, and bile acid metabolites. These pathways may contribute to chronic low-grade inflammation, insulin resistance, endothelial dysfunction, dyslipidemia, hepatic metabolic dysfunction, adipose tissue inflammation, adverse changes in body composition, sarcopenia, neuroinflammation, and urogenital microbial instability. Dietary patterns, fermentable fiber, physical activity, probiotics, synbiotics, phytoestrogens, and menopausal hormone therapy may influence components of this network, although the strength of evidence remains variable. Current data do not support the use of microbiome characteristics to select or dose hormone therapy. **Conclusions**: The gut microbiome may act as a dynamic mediator linking estrogen decline with metabolic and systemic aging after menopause. However, causality, individual variability, and clinical translation remain insufficiently established. Longitudinal studies integrating shotgun metagenomics, metabolomics, hormone measurements, standardized hormone therapy exposure, and clinical outcomes are needed to determine whether microbiome-derived biomarkers can support risk stratification and personalized interventions for healthier aging in postmenopausal women.

## 1. Introduction

Menopause is a major physiological transition characterized by the permanent cessation of menstruation resulting from loss of ovarian follicular activity and accompanied by substantial changes in sex hormone exposure [1,2]. Beyond reproductive function, the menopausal transition is associated with coordinated changes in metabolic, vascular, musculoskeletal, and neurocognitive physiology [1]. These changes are particularly relevant to cardiometabolic health, as the menopausal transition is accompanied by increases in visceral adiposity, adverse lipid changes, impaired insulin sensitivity, vascular dysfunction, and increasing cardiovascular risk, making midlife a critical window for the prevention of cardiometabolic and age-related disease [3,4].

Increasingly, this systemic perspective has expanded beyond the traditional endocrine paradigm to include the gut microbiome as a potential regulator of host physiology. The intestinal microbiota participates in bidirectional communication with endocrine, immune, and metabolic pathways and can influence energy homeostasis, inflammatory signaling, intestinal barrier function, and host metabolism through microbial metabolites [5,6]. Human studies suggest that menopause and estrogen deficiency may be associated with alterations in gut microbial composition and function; however, the direction, magnitude, and reproducibility of these changes remain incompletely defined [7,8]. Importantly, recent systematic review and meta-analytic evidence found no consistent differences in alpha diversity or the relative abundance of major bacterial phyla between hypoestrogenic and eugonadal women, underscoring substantial heterogeneity across studies and suggesting that menopause-associated effects may involve functional or taxon-specific changes that are not captured by broad compositional measures [9]. These findings do not exclude menopause-associated taxon-specific or functional microbial alterations and highlight the need to move beyond broad compositional measures when investigating the potential role of the gut microbiome in postmenopausal metabolic aging.

Central to this interaction is the estrobolome, defined as the collection of gut microbial genes whose products can metabolize estrogens [10,11]. Bacterial β-glucuronidases can deconjugate estrogen glucuronides in the intestine, facilitating reabsorption and potentially influencing systemic estrogen availability [11]. Bacterial sulfatases have also been proposed to contribute to estrogen deconjugation, although their role in enterohepatic estrogen recirculation was less clearly established in the evidence reviewed by Kwa et al. [11]. This provides a biologically plausible basis for a bidirectional relationship in which sex hormones can influence the gut microbial ecosystem, while microbial estrogen-metabolizing capacity may, in turn, affect host estrogen exposure [12]. Menopause-associated changes in the gut microbiome and estrobolome have therefore been proposed as potential contributors to interindividual variation in postmenopausal metabolic and endocrine phenotypes. However, the quantitative contribution of these microbial pathways to circulating estrogen concentrations and their causal relevance to clinical outcomes in postmenopausal women remain incompletely established [8].

Beyond estrogen metabolism, the gut microbiome exerts systemic effects through biologically active metabolites that link microbial activity with host physiology. Short-chain fatty acids (SCFAs) contribute to glucose homeostasis, intestinal barrier function, immune regulation, and energy metabolism [13], while secondary bile acids participate in metabolic signaling through receptors including FXR and TGR5 [14]. Microbially derived compounds such as lipopolysaccharide (LPS) and trimethylamine N-oxide (TMAO) have also been implicated in low-grade inflammation, endothelial dysfunction, and cardiovascular risk [15,16]. Alterations in these microbial pathways have been associated with metabolic and vascular phenotypes relevant to postmenopausal aging, although the extent to which menopause-related microbiome changes causally drive these outcomes remains uncertain.

Within this conceptual framework, metabolic syndrome represents a clinically relevant phenotype through which the convergence of visceral adiposity, insulin resistance, dyslipidemia, hypertension, and vascular dysfunction can be considered in relation to endocrine and microbial changes occurring after menopause [3]. Because the gut microbiome is responsive to diet, physical activity, and other environmental exposures, this axis represents a potentially modifiable component of postmenopausal metabolic health [5,6].

This concept is particularly relevant to the emerging field of precision nutrition, which recognizes substantial interindividual variation in dietary responses and metabolic phenotypes. Studies integrating dietary intake with microbiome and metabolic data have demonstrated that individuals can exhibit markedly different responses to the same nutritional exposure, supporting the rationale for more individualized nutritional strategies [17,18]. Whether microbiome-informed precision nutrition can improve clinically meaningful outcomes specifically in postmenopausal women remains an important unanswered question.

Accordingly, this narrative review examines the gut microbiome–estrogen axis in relation to body composition and cardiometabolic health during and after menopause, with particular attention to adiposity, skeletal muscle, insulin sensitivity, and hepatic and vascular metabolism. It also considers how nutrition and other interventions may influence these relationships. The conceptual framework presented in Figure 1 organizes the potential connections between estrogen decline, microbial functions, and metabolic outcomes, while distinguishing biological plausibility from demonstrated clinical effects. Brain and urogenital pathways are discussed as complementary aspects of endocrine–microbial interactions rather than as the primary outcomes of the review.

## 2. Materials and Methods

This narrative review was conducted to synthesize current evidence regarding the interactions between menopause, the gut microbiome, the estrobolome, microbial metabolites, and their potential contribution to microbiome-mediated metabolic aging, with particular emphasis on cardiometabolic health and emerging precision medicine approaches in postmenopausal women. A narrative review methodology was considered appropriate because the objective was to integrate mechanistic, translational, and clinical evidence across multiple interconnected disciplines, including endocrinology, microbiology, metabolism, nutrition, cardiovascular medicine, and women’s health, rather than to address a single narrowly defined clinical question.

The review primarily included studies published between January 2010 and April 2026. Earlier landmark publications were additionally considered when necessary to establish fundamental concepts, including menopause physiology, estrogen metabolism, the estrobolome, microbial metabolites, and the development of hormone replacement therapy. Current international clinical guidelines and consensus statements were also considered when relevant to clinical interpretation.

A literature search was performed using PubMed/MEDLINE, Scopus, and Web of Science. The search strategy combined controlled vocabulary, including Medical Subject Headings (MeSH) where applicable, with free-text terms related to menopause, postmenopause, gut microbiome, estrobolome, estrogen metabolism, microbial metabolites, metabolic aging, cardiometabolic health, nutrition, microbiome-targeted interventions, hormone replacement therapy, and precision medicine. Representative search terms included combinations of “menopause”, “postmenopause”, “gut microbiome”, “gut microbiota”, “estrobolome”, “estrogen metabolism”, “β-glucuronidase”, “short-chain fatty acids”, “trimethylamine N-oxide”, “bile acids”, “lipopolysaccharide”, “gut–brain axis”, “gut–muscle axis”, “metabolic syndrome”, “cardiovascular disease”, “metabolic dysfunction-associated steatotic liver disease”, “sarcopenia”, “precision nutrition”, “probiotics”, “synbiotics”, “phytoestrogens”, “hormone replacement therapy”, “metagenomics”, “metabolomics”, and “machine learning”. Additional relevant publications were identified by manual screening of the reference lists of included studies and relevant reviews.

Eligible publications included randomized controlled trials, prospective and retrospective cohort studies, case–control studies, cross-sectional and other observational studies, systematic reviews, meta-analyses, narrative reviews, clinical practice guidelines, and consensus statements addressing menopause-associated changes in the gut microbiome, estrobolome function, microbial metabolites, microbiome-targeted interventions, or precision medicine applications relevant to postmenopausal health. Because several areas addressed in this review, including pharmacomicrobiomics, microbiome-mediated metabolic aging, multi-omics integration, and machine learning, remain at an early translational stage, relevant mechanistic and experimental studies were also considered when they provided biological evidence directly informing the clinical framework discussed in the review. Studies conducted exclusively in pediatric populations or pregnancy, animal studies without clear translational relevance to the mechanisms discussed, conference abstracts, editorials, letters, and opinion articles without substantive scientific content were excluded.

Relevant publications were evaluated for study design, population characteristics, menopausal status or reproductive stage, microbiome-related exposures or interventions, microbial metabolites, clinical outcomes, mechanistic findings, and principal conclusions. For narrative synthesis, the evidence was organized into six thematic domains: (1) menopause-associated gut microbiome remodeling; (2) the estrobolome and microbial metabolites; (3) microbiome-mediated metabolic aging; (4) organ-specific consequences of microbiome dysregulation; (5) microbiome-targeted therapeutic strategies; and (6) precision medicine, multi-omics integration, and future clinical translation.

Given the substantial heterogeneity in study designs, populations, microbiome profiling methods, intervention protocols, and outcome measures, a quantitative meta-analysis was not attempted. Findings were therefore synthesized narratively within the predefined thematic domains. Greater interpretive weight was given to randomized controlled trials, systematic reviews and meta-analyses, clinical practice guidelines, and well-designed prospective cohort studies, whereas mechanistic and experimental evidence was used primarily to inform biological plausibility and was interpreted in the context of its translational limitations. Where evidence was indirect, preliminary, or derived predominantly from non-postmenopausal populations, this was considered when formulating the interpretation. The objective was to provide a clinically oriented synthesis of the available evidence, identify important knowledge gaps, and define research priorities for precision nutrition, pharmacomicrobiomics, and microbiome-informed precision medicine in postmenopausal women. For the tabulated evidence synthesis, representative sources were described according to study design, population, measured outcomes, and relevance to postmenopausal women. Clinical effects were distinguished from microbiome findings and evidence of microbial mediation. No formal certainty ratings were assigned, and inclusion in the table was not interpreted as a treatment recommendation.

No formal risk-of-bias assessment or certainty-of-evidence grading was performed because this study was designed as a narrative review rather than a systematic review or meta-analysis. Accordingly, the review does not claim to provide a systematic estimate of effect sizes or a formal hierarchy of evidence. Instead, the study design and evidentiary context of the cited literature were considered during narrative interpretation.

## 3. Menopause, Estrobolome and the Endocrine–Microbial Interface

Menopause represents far more than the cessation of ovarian cyclicity. It is a major biological transition characterized by progressive changes in ovarian function, circulating sex hormones, and multiple physiological systems [1,2]. Although vasomotor symptoms, sleep disturbances, and genitourinary symptoms remain the most recognizable clinical manifestations, the menopausal transition is also associated with changes in body composition, glucose and lipid metabolism, vascular function, bone health, and other determinants of healthy aging [1,3].

Growing evidence suggests that the gut microbiome may participate in this broader physiological remodeling. The intestinal microbiota interacts bidirectionally with endocrine, immune, and metabolic pathways through microbial metabolites, immune signaling, intestinal barrier function, and hormone metabolism [10,11,12]. As illustrated in the conceptual framework proposed in Figure 1, changes in the hormonal environment during the menopausal transition may interact with alterations in gut microbial composition and function, including changes in microbial pathways involved in estrogen metabolism [7,20]. This endocrine–microbial interface provides a biological basis for the concept of microbiome-mediated metabolic aging proposed in this review.

Importantly, the relationship between menopause and the gut microbiome appears to be bidirectional. Changes in sex-hormone exposure may influence microbial community structure and function, while gut microbial metabolism can modify the availability of circulating estrogens through deconjugation and enterohepatic recycling [11,12]. The magnitude and clinical consequences of these interactions are likely influenced by age, diet, metabolic status, medication use, and other host and environmental factors [20]. Accordingly, menopause-associated microbial changes should be viewed as one component of a broader endocrine–metabolic transition rather than as an isolated cause of metabolic dysfunction. This perspective also identifies the gut microbiome as a potentially modifiable component of postmenopausal health and provides a rationale for investigating dietary, lifestyle, and microbiome-directed interventions.

### 3.1. Hormonal Decline, Dysbiosis and Inflammaging

Estrogens exert pleiotropic effects extending beyond reproductive physiology and may contribute to intestinal barrier integrity, immune regulation, and host–microbiome interactions. Experimental and translational evidence indicates that estrogen signaling can influence intestinal epithelial and immune functions, while the gut microbiome participates in estrogen metabolism through microbial enzymes involved in estrogen deconjugation [12]. These observations provide a biological basis for bidirectional interactions between ovarian hormone status and the intestinal microbiome, although the extent to which changes in estrogen status directly determine specific microbial taxa or functional pathways in humans remains incompletely defined [6,12].

With the decline in ovarian hormone production, alterations in gut microbial composition and function have been reported across the menopausal transition. In a large metagenomic analysis of 2300 participants from the Hispanic Community Health Study/Study of Latinos, postmenopausal women showed altered microbial community composition and a trend toward lower diversity compared with premenopausal women after adjustment for age and multiple behavioral and demographic factors [7]. Importantly, menopause-associated differences included changes in microbial functional pathways and lower abundance of microbial β-glucuronidase genes, linking menopausal status to both microbial ecology and estrogen-related metabolic potential [7]. However, the magnitude and direction of microbiome changes across menopause are not fully consistent between populations, emphasizing the contribution of age, diet, adiposity, medication use, and other environmental factors [8,9,20,21].

Within the Study of Women’s Health Across the Nation (SWAN), markers of gut barrier dysfunction and microbial translocation increased across the menopause transition, with increases in fatty acid-binding protein 2 (FABP2), lipopolysaccharide-binding protein (LBP), and soluble CD14 (sCD14) [22]. Higher LBP and sCD14 levels were associated with higher C-reactive protein concentrations [22], providing human evidence for an association between the menopausal transition, intestinal barrier dysfunction, microbial translocation, and systemic immune activation.

These findings are biologically consistent with the broader concept of inflammaging, defined as a state of chronic, low-grade inflammation that contributes to age-related metabolic and cardiovascular disease [23]. The gut microbiome and its microbial products are recognized contributors to this inflammatory milieu, although the directionality and relative contribution of microbiome alterations versus host aging and metabolic dysfunction remain incompletely resolved [15].

In postmenopausal women, emerging evidence further links specific microbiome alterations with metabolic endotoxemia and adiposity. A recent metagenomic study of older postmenopausal women found that higher visceral adiposity was associated with increased abundance of Proteobacteria and elevated circulating markers of microbial endotoxemia, including lipopolysaccharide-binding protein [24]. These findings support a potential link between gut microbial composition, intestinal barrier-related immune activation, and metabolic dysfunction, although the cross-sectional design does not establish causality.

It should therefore be emphasized that the biological effects of menopause-associated microbiome remodeling are unlikely to depend solely on taxonomic composition. Changes in microbial metabolic activity, including SCFA production, bile acid transformation, endotoxin-related signaling, and estrogen deconjugation, may provide more functionally relevant links between the microbiome and systemic metabolic health [13,14,15,25]. These pathways are discussed in greater detail in the following section.

### 3.2. The Estrobolome: β-Glucuronidase Activity, Estrogen Recirculation and Inter-Individual Variability

Within the broader endocrine–microbial interface, the estrobolome refers to the collection of gut microbial genes and enzymatic activities involved in estrogen metabolism, particularly the deconjugation of estrogen metabolites. The concept was introduced by Plottel and Blaser to describe the capacity of the intestinal microbiome to influence estrogen metabolism and enterohepatic circulation [10]. Unlike ovarian estrogen production, which declines substantially after menopause, microbial estrogen-metabolizing capacity remains dynamic and may vary according to microbial composition, functional gene abundance, diet, medications, and other host and environmental factors [11,12,26].

The best-characterized mechanism involves bacterial β-glucuronidase activity. Following hepatic conjugation, estrogen metabolites are excreted into the intestine through bile [10,11]. Microbial β-glucuronidases can hydrolyze estrogen glucuronides, generating unconjugated metabolites that may subsequently undergo intestinal reabsorption and contribute to enterohepatic estrogen circulation [25]. Experimental and observational human data support a relationship between gut microbial composition, β-glucuronidase activity, and systemic estrogen metabolites, although the quantitative contribution of this pathway to circulating estrogen concentrations in postmenopausal women remains incompletely defined [25,27]. As illustrated in Figure 2, this process links hepatic estrogen conjugation and biliary excretion with microbial deconjugation, intestinal reabsorption, and subsequent systemic estrogen circulation. The estrobolome is therefore better understood as a functional property of the microbial community rather than a discrete group of bacterial taxa.

β-Glucuronidase activity is distributed across multiple bacterial lineages, and functional capacity depends on the abundance and activity of microbial genes rather than simply on the presence of individual species [25]. This functional variability provides a plausible biological explanation for inter-individual differences in microbial estrogen metabolism and may contribute to differences in systemic exposure to estrogen metabolites [11,27].

Importantly, human evidence now directly links menopause with alterations in the estrobolome. One study demonstrated altered gut microbial composition in postmenopausal compared to premenopausal women, together with a lower abundance of microbial β-glucuronidase genes [7]. These microbial differences were associated with cardiometabolic characteristics, supporting an association between menopausal status, microbial estrogen metabolism, and metabolic health [7].

However, the extent to which altered estrobolome activity directly determines systemic estrogen availability after menopause remains uncertain. Most available human studies are cross-sectional, and the relationship between microbial β-glucuronidase activity, circulating estrogen metabolites, and clinical outcomes is likely to be influenced by age, adiposity, diet, medication exposure, and host hepatic metabolism [7,21,27]. Accordingly, the estrobolome should currently be considered a potential modulator of estrogen metabolism rather than an established independent determinant of postmenopausal estrogen status.

This distinction is particularly relevant to hormone replacement therapy (HRT). Oral estrogen undergoes hepatic first-pass metabolism and enterohepatic cycling, providing a theoretical route through which intestinal microbial metabolism could influence the disposition of estrogen conjugates [10,11,28]. Transdermal estrogen, in contrast, bypasses gastrointestinal absorption and hepatic first-pass metabolism [28,29]. However, direct clinical evidence demonstrating that inter-individual variation in estrobolome activity materially alters HRT pharmacokinetics or clinical response is currently lacking. The potential interaction between estrobolome function and HRT therefore remains a mechanistically plausible but clinically unvalidated hypothesis, rather than an established basis for route or dose selection.

The broader concept is consistent with the emerging field of pharmacomicrobiomics, which examines how inter-individual variation in gut microbial composition and function contributes to drug absorption, metabolism, pharmacokinetics, pharmacodynamics, efficacy, and toxicity [30,31,32]. Although convincing examples exist for several drug classes, including drugs undergoing microbial deconjugation or biotransformation [30,31,32], comparable clinical evidence for menopausal hormone therapy is currently insufficient.

Consequently, estrobolome profiling represents a promising component of future precision medicine but is not yet ready for routine clinical application. Prospective studies combining shotgun metagenomics, estrogen metabolomics, pharmacokinetic measurements, and standardized HRT exposure are needed to determine whether microbial estrogen-metabolizing capacity can predict hormone exposure, treatment response, or adverse effects. Similarly, randomized trials are required to establish whether dietary, probiotic, prebiotic, or other microbiome-directed interventions can reproducibly modify estrobolome activity and produce clinically meaningful changes in postmenopausal outcomes.

## 4. Microbial Metabolite Imbalance as a Driver of Microbiome-Mediated Metabolic Aging

If the estrobolome represents an important interface between the gut microbiome and estrogen metabolism, microbial metabolites constitute major functional mediators through which intestinal microbial activity can influence systemic physiology. Rather than representing passive end products of bacterial metabolism, these molecules participate in signaling between the microbiota and host immune, metabolic, endocrine, and cardiovascular pathways [19,33]. Microbiota-derived metabolites and microbial components can influence distant organs, including the liver, adipose tissue, skeletal muscle, vasculature, and brain, through effects on cellular signaling, immune regulation, energy metabolism, and epithelial barrier function [19,33].

During reproductive aging, alterations in gut microbial composition have been accompanied by changes in microbial functional potential and circulating or fecal metabolite profiles. Postmenopausal women showed differences in gut microbial composition and functional pathways compared with premenopausal women, and these microbiome features were associated with cardiometabolic characteristics [7]. A study of 44 women with menopausal symptoms, grouped by follicle-stimulating hormone concentration, identified differences in gut microbiota and fecal metabolic pathways involving lipids and amino acids; its cross-sectional design limits causal interpretation [34].

These findings suggest that menopause-associated changes in the gut microbiome may affect not only which microorganisms are present but also the metabolites they produce. However, it remains unclear whether menopause itself causes consistent changes in microbial metabolite profiles, as these profiles are also influenced by age, diet, adiposity, medication use, physical activity, and other host factors. Accordingly, microbial metabolite alterations should be considered potential mediators of the relationship between reproductive aging and metabolic health rather than established causal determinants of postmenopausal metabolic aging.

Among the microbial metabolites and microbial-derived products most relevant to cardiometabolic physiology, SCFAs, secondary bile acids, trimethylamine N-oxide (TMAO), and LPS-related pathways are among the best studied [13,14,15,16]. These pathways act through distinct but interconnected mechanisms involving glucose and lipid metabolism, intestinal barrier function, immune activation, vascular biology, and systemic inflammation [13,14,15,19].

### 4.1. Short-Chain Fatty Acids and Insulin Sensitivity

SCFAs, primarily acetate, propionate, and butyrate, are major products of the anaerobic fermentation of dietary fiber by the gut microbiota [13]. Their production depends on the metabolic activity of fiber-fermenting microbial communities, including taxa within the genera *Faecalibacterium*, *Roseburia*, and *Eubacterium*, with additional contributions arising through metabolic cross-feeding [13,35]. Beyond their role as fermentation products, SCFAs function as signaling molecules linking dietary substrates and microbial activity with intestinal barrier function, immune regulation, and host metabolic physiology [13,35].

SCFAs exert their biological effects through several complementary mechanisms. Activation of the G protein-coupled receptors FFAR2 (GPR43) and FFAR3 (GPR41) on enteroendocrine and immune cells can modulate the secretion of glucagon-like peptide-1 (GLP-1) and peptide YY (PYY), thereby influencing glucose homeostasis and energy regulation [13]. Butyrate also serves as a major energy substrate for colonocytes and contributes to epithelial barrier maintenance [13,35]. In addition, butyrate and propionate can inhibit histone deacetylases, providing an epigenetic mechanism through which microbial metabolites influence inflammatory and immune pathways [35].

In postmenopausal women, evidence linking SCFA alterations to metabolic phenotypes remains limited but increasingly relevant. A cross-sectional study of obese postmenopausal women identified associations between gut microbial composition, fecal SCFA concentrations, visceral adiposity, and glucose-related metabolic parameters, with mediation analyses suggesting that propionate may contribute to some microbiota–glucose relationships [36]. These findings support a potential role for SCFA-related microbial metabolism in postmenopausal cardiometabolic physiology, although their observational design does not establish causality.

Interventional evidence is also emerging. A post hoc analysis of a randomized, double-blind, placebo-controlled trial included 56 postmenopausal women with obesity and reported increases in fecal acetate, butyrate, and valerate concentrations in the high-dose multispecies probiotic group [37]. These findings indicate that probiotic supplementation can modify fecal SCFA concentrations in this population. However, fecal concentrations do not directly quantify intestinal SCFA production or systemic availability, and the long-term clinical implications remain uncertain.

From a clinical perspective, dietary strategies that increase fermentable fiber intake represent a biologically plausible approach to enhancing endogenous SCFA production [13,35]. Prebiotics and selected probiotic interventions may additionally modify SCFA availability, although effects are dependent on the specific substrate, microbial community, strain, dose, and host metabolic context [35,38]. This provides a mechanistic basis for precision nutrition approaches in which dietary interventions are evaluated according to individual microbiome and metabolic characteristics rather than assumed to produce uniform responses. Nevertheless, prospective longitudinal studies integrating dietary intake, microbiome composition, microbial metabolomics, and metabolic outcomes are required to determine whether sustained modulation of SCFA pathways can alter the trajectory of metabolic aging after menopause.

### 4.2. Lipopolysaccharides, Endotoxemia and Chronic Inflammation

LPS, a major structural component of the outer membrane of Gram-negative bacteria, is one of the best-characterized microbial products linking intestinal barrier dysfunction with systemic inflammation. Under physiological conditions, the intestinal epithelial barrier limits the passage of microbial components into the circulation. Increased intestinal permeability may facilitate greater exposure to LPS and other microbial products, contributing to a state commonly described as metabolic endotoxemia [15,39]. Experimental and clinical studies have associated metabolic endotoxemia with adipose tissue inflammation, impaired insulin signaling, and cardiometabolic dysfunction, although the magnitude and clinical significance of circulating LPS depend strongly on the assay and biological context [15,33,39].

LPS exerts its principal inflammatory effects through activation of Toll-like receptor 4 (TLR4), together with the CD14/MD-2 receptor complex, triggering downstream NF-κB and related inflammatory signaling pathways [15,33]. Activation of these pathways promotes the production of pro-inflammatory mediators, including TNF-α, IL-6, and IL-1β [15,33]. In metabolic tissues, persistent innate immune activation can interfere with insulin signaling, promote adipose tissue inflammation, and contribute to hepatic and vascular dysfunction [15,39]. These mechanisms provide a biologically plausible link between altered intestinal barrier function, microbial exposure, and systemic metabolic inflammation.

In the context of menopause, estrogen decline may interact with these pathways through effects on immune regulation, intestinal physiology, and metabolic homeostasis [3,12]. However, the extent to which menopause-associated microbiome remodeling directly increases circulating LPS or intestinal permeability in humans remains insufficiently characterized. Therefore, rather than considering LPS-mediated endotoxemia an established consequence of menopause, it is more appropriate to view it as a potential mechanistic link between intestinal barrier dysfunction, chronic inflammation, and postmenopausal metabolic deterioration. This distinction is important because age, adiposity, diet, physical activity, medication use, and metabolic disease independently influence both intestinal permeability and inflammatory status [6,15].

The potential clinical relevance of this pathway lies in the possibility that interventions improving intestinal barrier function and modifying microbial activity may reduce systemic inflammatory exposure. Dietary fiber, overall dietary quality, and selected microbiome-targeted interventions can influence intestinal microbial composition and metabolite production [40,41,42], but evidence that such interventions specifically reduce LPS-mediated endotoxemia and improve long-term cardiometabolic outcomes in postmenopausal women remains limited. Accordingly, future studies should combine standardized assessment of gut permeability, circulating microbial products, microbiome composition and function, and metabolic outcomes to determine whether endotoxemia-related biomarkers have value for risk stratification or treatment monitoring in postmenopausal women.

### 4.3. TMAO, Bile Acids and Cardiometabolic Risk

TMAO is one of the most extensively studied gut microbiota-derived metabolites in relation to cardiovascular risk. TMAO is generated through a two-step pathway in which gut microorganisms convert dietary substrates containing choline, phosphatidylcholine, and L-carnitine to trimethylamine (TMA), which is subsequently oxidized in the liver by flavin-containing monooxygenase 3 (FMO3) [16,43,44]. This pathway illustrates the interaction between dietary substrate availability, microbial metabolism, and host hepatic function and provides a mechanistic basis for inter-individual differences in circulating TMAO concentrations.

Experimental studies have implicated TMAO in several pathways relevant to atherosclerosis and thrombosis, including altered cholesterol metabolism and impaired reverse cholesterol transport [43,44], as well as increased platelet reactivity [45]. In humans, higher circulating TMAO concentrations have been associated with increased cardiovascular risk in prospective cohort studies [16], although the extent to which TMAO is a causal mediator rather than a marker of dietary, metabolic, renal, or microbial factors remains an active area of investigation.

The relevance of TMAO to postmenopausal health is biologically plausible but remains incompletely characterized. Menopause-associated changes in microbial composition, dietary patterns, body composition, and cardiometabolic status may influence TMA production and circulating TMAO concentrations; however, direct evidence demonstrating a menopause-specific increase in TMAO or a causal pathway linking estrogen decline to altered TMAO metabolism remains limited. Importantly, inter-individual responses to dietary precursors may be substantial. In a randomized crossover study of overweight postmenopausal women, consumption of two whole eggs per day increased circulating choline and betaine but did not significantly increase plasma TMAO or alter overall gut microbiome composition, illustrating the complexity of the diet–microbiome–TMAO relationship [46].

Bile acids represent a second major class of microbiome-modified metabolites with important roles in metabolic regulation. Primary bile acids synthesized in the liver undergo microbial deconjugation and further biotransformation in the intestine, generating secondary bile acids that participate in host signaling through the farnesoid X receptor (FXR) and the G protein-coupled bile acid receptor 1 (TGR5) [14]. These pathways influence bile acid synthesis and transport, glucose and lipid metabolism, energy expenditure, and enteroendocrine signaling, including GLP-1 secretion [14].

Alterations in gut microbial composition can therefore modify the intestinal bile acid pool and downstream host signaling. Changes in bile acid metabolism have been implicated in metabolic disorders characterized by dyslipidemia, impaired glucose homeostasis, and hepatic metabolic dysfunction [14]. In postmenopausal women, emerging evidence suggests that menopause-associated changes in the gut microbiome and host metabolism may be accompanied by alterations in microbial and host metabolic pathways, but the specific contribution of bile acid signaling to postmenopausal cardiometabolic risk remains insufficiently defined [6,47].

Taken together, TMAO and bile acid pathways provide biologically plausible links between dietary exposure, microbial metabolism, and cardiometabolic physiology. Their clinical relevance in postmenopausal women, however, should not yet be overstated. Prospective studies integrating menopausal staging, dietary assessment, gut microbiome profiling, targeted metabolomics, and cardiovascular outcomes are needed to determine whether TMAO and microbiome-derived bile acid signatures improve cardiovascular risk prediction beyond established clinical risk factors. Such studies may also clarify whether individual metabolic responses can be used to guide precision nutrition or microbiome-targeted interventions during the postmenopausal transition.

## 5. Organ Crosstalk in Postmenopausal Aging: One Network, Multiple Windows

The mechanisms described in the preceding sections, including alterations in estrogen–microbiome interactions, intestinal barrier function, SCFA metabolism, TMAO production, and bile acid signaling, do not act exclusively within individual organ systems. Rather, they form interconnected biological pathways through which gut microbial activity may influence multiple tissues via shared metabolic, inflammatory, endocrine, and immune mechanisms [6,15,19]. This systems-level perspective provides a framework for understanding why menopause-associated metabolic disturbances frequently extend beyond a single organ and manifest across several physiological domains. These interconnected pathways may contribute to distinct but overlapping phenotypes across organ systems. In the cardiovascular system, relevant manifestations include endothelial dysfunction, vascular inflammation, and atherosclerotic risk; in the liver, altered lipid handling and hepatic steatosis; in skeletal muscle, impaired glucose metabolism and loss of muscle mass and function; in the brain, neuroinflammatory and metabolic changes associated with cognitive and affective outcomes; and in the urogenital tract, epithelial and microbial alterations associated with estrogen deficiency. Importantly, the strength of evidence linking gut microbial alterations to these outcomes differs substantially across organ systems, and associations should not be interpreted as evidence that microbiome dysfunction is the sole or primary cause of postmenopausal disease.

Accordingly, the concept of microbiome-mediated metabolic aging proposed in this review should be regarded as an integrative framework rather than a proven single causal pathway. It emphasizes the potential contribution of gut microbial ecology and metabolism to the biological processes accompanying postmenopausal aging, while recognizing the substantial influence of age, adiposity, diet, physical activity, medication exposure, genetics, and other host factors.

The following subsections prioritize tissue-specific pathways relevant to body composition and cardiometabolic health. The discussions of brain and urogenital physiology provide complementary context for the broader endocrine–microbial network, with their relevance and evidentiary limitations considered separately.

### 5.1. Cardiovascular System—Arterial Stiffness, Atherosclerosis and Hypertension

The cardiovascular system represents an important target of postmenopausal cardiometabolic aging, where endocrine changes may interact with alterations in gut microbial composition and function. Premenopausal women benefit from several vasoprotective effects of estrogens, including effects on endothelial nitric oxide signaling, vascular tone, arterial compliance, and inflammatory pathways [1,3]. Following menopause, the decline in estrogen exposure is accompanied by adverse changes in vascular and cardiometabolic risk factors [3], while alterations in the gut microbiome and its metabolites may represent additional mechanisms contributing to this phenotype [6,15].

Microbial metabolites may influence vascular function through several complementary pathways. Metabolic endotoxemia can activate TLR4-dependent inflammatory signaling and has been linked to impaired insulin signaling and endothelial dysfunction [15,39]. Elevated circulating TMAO has been associated with pro-atherogenic processes, including altered cholesterol metabolism and platelet activation [43,44,45], and higher circulating TMAO concentrations have been associated with increased cardiovascular risk in prospective human studies [16]. In contrast, SCFAs participate in immune, metabolic, and vascular signaling, although their direct effects on cardiovascular outcomes in postmenopausal women remain insufficiently characterized [13]. A potential point of convergence is the interaction between reduced estrogen-dependent vascular protection and increased exposure to gut-derived inflammatory signals. Human observations of increased intestinal barrier dysfunction and microbial translocation markers across the menopause transition support the plausibility of this connection [22]. Such exposure could amplify vascular inflammation in the setting of declining estrogen signaling, although a causal sequence linking menopause-associated microbiome remodeling to vascular dysfunction has not been demonstrated in women [3,15].

Hypertension is another major component of postmenopausal cardiometabolic risk [3]. Experimental and observational evidence suggests that the gut microbiome may participate in blood pressure regulation through microbial metabolites, immune signaling, intestinal barrier function, and interactions with host metabolic pathways [48]. However, the extent to which microbiome alterations independently contribute to the increase in blood pressure observed after menopause remains uncertain. In postmenopausal women, microbiome-related mechanisms may therefore interact with estrogen deficiency and established cardiovascular risk factors to influence vascular aging.

From a clinical perspective, microbiome-derived metabolites, including SCFAs, LPS-related markers, and TMAO, represent potential biomarkers of cardiometabolic risk and possible targets for future intervention [13,15,16]. Nevertheless, the current evidence does not support their use as replacements for established cardiovascular risk assessment. Prospective studies in postmenopausal women are needed to determine whether microbiome profiling or circulating microbial metabolites provide incremental predictive value beyond conventional risk factors and whether they can identify women who respond preferentially to dietary, pharmacological, or hormone-based interventions.

### 5.2. Liver—NAFLD, Dyslipidemia and Hepatic Insulin Resistance

The liver is a central metabolic organ in which endocrine, metabolic, and microbial pathways converge during the menopausal transition. Estrogen deficiency is associated with adverse changes in hepatic lipid metabolism and insulin sensitivity, including increased hepatic lipid accumulation and altered lipoprotein metabolism [47,49]. Postmenopausal status is associated with a greater risk of progressive fibrosis in metabolic dysfunction-associated steatotic liver disease (MASLD), which replaces the former non-alcoholic fatty liver disease (NAFLD) terminology [50]. Although gut microbiome alterations have been associated with hepatic steatosis and metabolic dysfunction [15], the extent to which menopause-associated microbial remodeling independently contributes to this increased risk remains incompletely defined.

Gut-derived microbial products may influence liver physiology through the gut–liver axis. Increased intestinal permeability can facilitate the delivery of microbial components, including LPS, to the portal circulation, where they may activate hepatic innate immune pathways and contribute to inflammation and metabolic dysfunction [15]. Microbial modification of bile acids represents an additional pathway linking the intestine and liver. Primary bile acids undergo microbial transformation into secondary bile acids that participate in host signaling through receptors including the farnesoid X receptor (FXR) and TGR5, which regulate bile acid homeostasis and multiple aspects of glucose and lipid metabolism [14]. Alterations in these pathways have been associated with metabolic dysfunction and hepatic steatosis, although their specific contribution to postmenopausal liver disease remains to be established.

Experimental evidence provides a more direct connection between estrogen deficiency, microbial alterations, and hepatic dysfunction. In ovariectomized mice fed a high-fat diet, estrogen deficiency was accompanied by altered gut microbial composition, reduced butyrate content, impaired expression of intestinal barrier-related proteins, and aggravated hepatic steatosis [49]. Fecal microbiota transplantation or butyrate supplementation attenuated hepatic steatosis in this model [49]. These findings support a potential pathway in which reduced estrogen signaling and altered microbial metabolism jointly compromise intestinal homeostasis and contribute to hepatic lipid dysregulation. However, these intervention findings were obtained in mice and do not establish an equivalent causal pathway in naturally postmenopausal women.

In humans, longitudinal observations across the menopause transition have linked declining ovarian function with increases in markers of intestinal barrier dysfunction and microbial translocation [22]. When considered alongside experimental evidence on metabolic endotoxemia [39], these findings suggest a possible connection between the menopausal endocrine transition, greater exposure to gut-derived inflammatory signals, and hepatic metabolic dysfunction. Microbial bile acid transformation provides a complementary route through which changes in gut microbial function could modify FXR- and TGR5-dependent metabolic signaling [14]. These pathways may converge on hepatic lipid handling and insulin sensitivity, but their relative contributions to postmenopausal MASLD have not been established.

From a clinical perspective, the gut–liver axis represents a potential target for nutritional and microbiome-directed interventions in women after menopause. Dietary quality, physical activity, and weight management remain the established foundations of MASLD prevention and management [50], whereas microbiome-targeted strategies are promising but require further clinical validation. Prospective studies integrating menopausal status, dietary exposure, gut microbiome composition, microbial metabolites, and objectively assessed hepatic outcomes are needed to determine whether microbiome-derived markers provide additional value for risk stratification or personalized treatment.

### 5.3. Muscle—Sarcopenia and Insulin Resistance

Skeletal muscle undergoes important metabolic and functional changes during aging and the menopausal transition. Estrogen contributes to skeletal muscle homeostasis through effects on muscle protein metabolism, mitochondrial function, and insulin sensitivity [1,51]. Following menopause, declining estrogen exposure is associated with changes in muscle mass, strength, and metabolic function, contributing to an increased susceptibility to sarcopenia and insulin resistance [1,6].

The gut–muscle axis has emerged as a potential pathway linking intestinal microbial activity with skeletal muscle metabolism. Microbial metabolites, particularly SCFAs, may influence muscle physiology through effects on energy metabolism, immune regulation, mitochondrial function, and insulin signaling [13,51,52]. Conversely, aging-related changes in muscle metabolism and physical activity may also influence the gut microbiome, supporting a bidirectional relationship between microbial and muscle health [51,52]. However, evidence directly demonstrating that menopause-associated changes in the gut microbiome cause sarcopenia in humans remains limited.

Inflammatory signaling may represent an additional link between gut microbial alterations and muscle dysfunction. Chronic low-grade inflammation and increased concentrations of inflammatory mediators such as IL-6 and TNF-α have been associated with impaired muscle anabolism, insulin resistance, and loss of muscle function during aging [23,52]. Gut microbiome alterations may contribute to this inflammatory environment through microbial metabolites and intestinal barrier dysfunction [15], although the relative contribution of these pathways to postmenopausal sarcopenia remains uncertain.

These pathways may converge when declining estrogen signaling and gut-derived inflammatory exposure jointly affect skeletal muscle metabolism. Reduced estrogen-dependent support of muscle homeostasis may increase susceptibility to metabolic dysfunction, while microbial products reaching the circulation could reinforce inflammatory pathways that impair insulin signaling and anabolic responses [1,15,52]. Altered SCFA availability provides a complementary potential link through effects on immune regulation and energy metabolism [13,52]. This interaction remains a mechanistic hypothesis: current evidence does not establish that menopause-associated microbiome changes mediate the loss of muscle mass or strength in women.

From a clinical perspective, physical activity and nutritional support are central strategies discussed in the broader sarcopenia literature [51,52]. Exercise may also influence gut microbial composition and metabolic activity, providing a potential point of convergence between musculoskeletal and microbiome health [53]. Dietary fiber and adequate protein intake may further support metabolic and muscle health [35,52], although evidence that microbiome-targeted interventions specifically prevent or reverse sarcopenia in postmenopausal women remains insufficient. Prospective studies integrating physical activity, dietary exposure, muscle phenotyping, gut microbiome profiling, and metabolomics are needed to determine whether the gut–muscle axis can be translated into clinically useful prevention or treatment strategies.

### 5.4. Adipose Tissue—Visceral Adiposity and Metabolic Inflammation

Body composition changes during menopause involve both adipose tissue and skeletal muscle. Longitudinal data from the Study of Women’s Health Across the Nation showed an acceleration of fat mass gain and a decline in lean mass during the menopausal transition, without a corresponding acceleration in body weight gain [54]. Thus, body weight alone may obscure changes in the relative proportions of fat and lean tissue. Menopause-associated abdominal fat accumulation is particularly relevant to cardiometabolic health, although chronological aging, physical activity, and dietary intake also contribute to these changes [3].

Direct evidence linking the gut microbiome to visceral adiposity in postmenopausal women comes from a study of older women selected for low or high visceral adipose tissue area measured by dual-energy X-ray absorptiometry. Higher visceral adiposity was associated with greater Proteobacteria abundance, while circulating lipopolysaccharide-binding protein was positively associated with visceral adipose tissue area [24]. These observations connect regional adiposity with microbial composition and markers of endotoxin-related immune activation. However, the cross-sectional design and advanced age of the participants limit conclusions about causality and the contribution of the menopausal transition itself.

A potential mechanistic connection is that estrogen-related changes in fat distribution may interact with gut-derived inflammatory signals to amplify adipose tissue dysfunction. Experimental evidence indicates that sustained LPS exposure can promote adipose inflammation and insulin resistance [39]. Altered microbial SCFA metabolism may also influence energy regulation and immune signaling, although the direction and magnitude of these effects depend on the metabolite and host context [13,35]. Together, these pathways provide a plausible connection between endocrine changes, microbial activity, and adipose metabolic dysfunction, rather than evidence that a specific menopausal microbiome causes obesity.

Adiposity should therefore be considered both a potential outcome and a source of confounding in studies of the microbiome–estrogen axis. Future longitudinal studies should assess regional fat distribution alongside lean mass, hormone concentrations, dietary intake, and microbial functions to determine whether microbiome alterations precede or follow changes in body composition. This distinction is essential for evaluating whether microbiome-targeted interventions can improve adiposity-related outcomes beyond established nutritional and physical activity strategies.

### 5.5. Brain—Neuroinflammation, Cognitive Decline and Mental Health

The gut–brain axis represents a bidirectional communication network through which the gut microbiome may influence central nervous system function through neural, endocrine, immune, and metabolic pathways [55]. During the menopausal transition, declining estrogen exposure may interact with brain aging and changes in mood and cognition. Estrogens contribute to neuronal and vascular homeostasis through effects on synaptic plasticity, neurotransmitter signaling, and neuroinflammatory pathways [1]. At the same time, alterations in the gut microbiome may modify systemic inflammatory and metabolic signals reaching the central nervous system, providing a potential link between endocrine aging, microbial remodeling, and brain health [6,55].

Emerging evidence indicates that gut microbial alterations may influence neuroimmune signaling through intestinal barrier function, systemic inflammation, microbial metabolites, and tryptophan metabolism [55,56]. Microbially derived SCFAs and other metabolites can affect immune and neuronal pathways [55], while alterations in tryptophan metabolism may influence the balance between serotonin synthesis and kynurenine-pathway metabolites [56]. However, much of the mechanistic evidence linking these pathways to neuroinflammation and cognitive dysfunction derives from experimental models, and the clinical significance of these mechanisms in postmenopausal women remains incompletely established.

A potential connection between these processes is that declining estrogen signaling may alter the neural and immune context in which gut-derived signals act. Changes in intestinal barrier function and microbial metabolism could modify peripheral inflammatory signaling and tryptophan metabolism, thereby interacting with estrogen-sensitive pathways involved in neuronal function and neuroimmune regulation [12,55,56]. This convergence offers a mechanistic hypothesis linking endocrine and microbial changes, but it has not been established as a causal explanation for cognitive or affective outcomes in postmenopausal women.

From a clinical perspective, the gut–brain axis represents a potential avenue for future nutritional and microbiome-directed approaches to brain health after menopause. However, evidence supporting microbiome-targeted interventions for the prevention or treatment of cognitive decline or mental health disorders specifically in postmenopausal women remains limited. Prospective studies combining longitudinal microbiome profiling, metabolomics, standardized cognitive and psychological assessments, and, where appropriate, neuroimaging are needed to determine whether microbiome-related biomarkers provide clinically meaningful information beyond established neurological and cardiovascular risk factors.

### 5.6. Urogenital Tract—Vaginal Dysbiosis and Local Immunity

The urogenital tract represents a distinctive component of the menopausal microbiome because it is directly influenced by estrogen deficiency and contributes to local epithelial and immune homeostasis. In premenopausal women, the vaginal microbiome is typically dominated by *Lactobacillus* species, particularly *Lactobacillus crispatus*, *L. iners*, *L. gasseri*, and *L. jensenii* [57]. These organisms contribute to maintenance of a low vaginal pH through lactic acid production and are associated with colonization resistance against potential pathogens [58]. Estrogen supports this ecosystem by promoting glycogen accumulation in the vaginal epithelium, thereby providing substrates that favor Lactobacillus growth [58].

Following menopause, declining estrogen levels are associated with reduced vaginal glycogen availability, decreased *Lactobacillus* dominance, increased vaginal pH, and greater representation of anaerobic bacterial taxa [58,59]. These changes are characteristic of the vaginal microbial remodeling observed after menopause and may contribute to the pathophysiology of genitourinary syndrome of menopause (GSM). Vaginal microbial alterations have also been associated with epithelial disruption and local inflammatory responses, while reduced *Lactobacillus* dominance may decrease resistance to urogenital pathogens [58].

Evidence also suggests an interaction between the vaginal and urinary microbial environments. Changes in the postmenopausal vaginal microbiome, particularly loss of *Lactobacillus* dominance, may reduce colonization resistance against uropathogens and contribute to susceptibility to recurrent urinary tract infection (UTI) in some women [60]. However, the extent to which microbiome alterations independently determine recurrent UTI risk remains incompletely established, given the contribution of estrogen deficiency, urinary tract anatomy, prior antibiotic exposure, and other host factors.

In this compartment, the endocrine–microbial connection is primarily local: reduced estrogen stimulation alters the vaginal epithelium and substrate availability, which may reduce support for Lactobacillus-dominated communities and their contribution to an acidic vaginal environment [58]. Associations between menopausal status, vaginal community composition, and vulvovaginal atrophy support this local relationship, although they do not establish microbial changes as the sole cause of symptoms [59]. The vaginal microbiome should therefore be distinguished from the intestinal microbiome; a gut-mediated contribution to these local postmenopausal changes remains insufficiently established.

From a clinical perspective, local vaginal estrogen is an established treatment for GSM [28,29], whereas microbiome-directed approaches, including probiotics and other microbial interventions, remain under investigation. Although restoration of Lactobacillus dominance represents a biologically plausible therapeutic target, evidence that microbiome manipulation alone can sustainably prevent recurrent UTI or modify clinically important urogenital outcomes remains insufficient. Future prospective studies should therefore evaluate vaginal microbiome composition alongside estrogen exposure, epithelial biomarkers, inflammatory mediators, and clinical outcomes to determine whether microbiome profiles can identify women who may benefit from adjunctive microbiome-directed interventions.

## 6. Interventions Targeting the Microbiome–Metabolism Axis

The conceptual framework presented in the preceding sections suggests that the gut microbiome may represent a modifiable component of metabolic health during and after menopause. Human studies have reported menopause-associated differences in microbial functional potential and increases in markers of intestinal barrier dysfunction and microbial translocation [7,22]. However, the extent to which these alterations can be therapeutically modified in postmenopausal women, and whether such modification translates into clinically meaningful metabolic or cardiovascular benefits, remains incompletely established.

This section reviews five major categories of interventions relevant to the microbiome–metabolism axis: dietary patterns and fermentable fiber, physical activity, probiotics and synbiotics, phytoestrogens, and menopausal hormone therapy (MHT). These interventions differ substantially in their mechanisms, level of evidence, and degree of clinical validation. Accordingly, they are discussed not as equivalent microbiome-targeted therapies, but according to the available evidence regarding microbiome modulation, metabolic effects, clinical outcomes, and translational relevance.

Table 1 summarizes representative evidence for these interventions, distinguishing the study populations and measured outcomes from proposed microbiome mechanisms and their applicability to postmenopausal care.

Figure 3 shows the conceptual framework illustrating microbiome-relevant interventions in postmenopausal women. Lifestyle, nutritional, microbial, and hormonal interventions may influence the gut microbiome and related microbial functions, including microbial diversity, estrobolome activity, and microbial metabolite production. These changes may contribute to metabolic and systemic effects across multiple organ systems and may ultimately support individualized approaches to prevention and management. Integration of microbiome profiling with metagenomic and metabolomic approaches may provide a basis for future precision nutrition and risk stratification.

### 6.1. Dietary Patterns and Fermentable Fiber

Diet is one of the major determinants of gut microbiome composition and function, with measurable changes in microbial communities occurring within days to weeks following dietary modification [68]. In postmenopausal women, dietary interventions have attracted increasing attention because they may partially counteract microbiome-mediated metabolic aging by promoting beneficial microbial taxa, enhancing microbial metabolite production, and reducing low-grade inflammation. Among the available dietary patterns, the Mediterranean diet has evidence supporting microbial changes and cardiovascular benefits from separate studies [61,62].

The Mediterranean diet provides fermentable substrates through vegetables, fruits, legumes, whole grains, and nuts. In the NU-AGE dietary intervention, greater adherence to a Mediterranean-style diet was associated with changes in gut microbial composition, and diet-responsive microbial features were associated with lower inflammatory markers and reduced frailty [61]. This study involved older adults of both sexes and did not establish a menopause-specific effect. Evidence for cardiovascular event reduction comes from a separate trial, PREDIMED [62], and should not be interpreted as proof that its cardiovascular benefits were mediated by the microbiome.

Fermentable fiber deserves particular attention as a microbiome-targeted nutritional strategy. In a randomized, double-blind, placebo-controlled crossover trial in adults with mild constipation, inulin supplementation increased the relative abundance of Bifidobacterium [40]. Similarly, a randomized, placebo-controlled crossover trial in adults with type 2 diabetes found that inulin-type fructans increased bifidobacterial abundance and fecal concentrations of total SCFAs, acetate, and propionate [41]. These findings support a bifidogenic effect of specific inulin-type fructans, rather than a uniform effect of all dietary fibers. Neither trial specifically targeted postmenopausal women, and these microbial changes should not be interpreted as proof of improved intestinal barrier function or cardiometabolic outcomes in this population. The optimal substrate, dose, and duration for postmenopausal women remain to be established.

Dietary protein sources also provide a context for diet–microbiome interactions. Legumes, nuts, and other plant foods supply fiber and bioactive compounds alongside protein [68]. In a human L-carnitine challenge study, TMAO responses differed between omnivores and vegans or vegetarians, supporting an interaction between habitual diet and microbial metabolism [44]. These findings do not establish that replacing animal protein with plant protein reduces cardiovascular risk through TMAO lowering in postmenopausal women. Both protein adequacy and the overall dietary pattern should therefore be considered, while microbial mediation of clinical benefits remains to be established.

From a clinical perspective, dietary modification represents the most accessible strategy for targeting the gut microbiome in postmenopausal women. A Mediterranean-style dietary pattern can complement conventional approaches to reducing cardiometabolic risk [62], while fermentable fiber can modify selected microbial features [40,41]. Whether these changes attenuate microbiome-mediated metabolic aging after menopause remains uncertain. However, well-designed longitudinal trials integrating dietary assessment with metagenomic, metabolomic, and clinical outcomes are still needed to identify the most effective precision nutrition strategies for this population.

### 6.2. Physical Activity and the Gut–Muscle Axis

Physical activity is an established strategy for supporting postmenopausal metabolic and musculoskeletal health [3,63,64]. Its effects on the gut microbiome are still being characterized. In a six-week endurance exercise intervention in lean adults and adults with obesity, microbial and fecal SCFA responses depended on obesity status and largely reversed after exercise stopped [53]. This study was not specific to postmenopausal women and does not establish a consistent microbial response to both aerobic and resistance exercise in this population.

The gut–muscle axis describes the bidirectional communication between skeletal muscle and the intestinal microbiome [51,52]. Exercise-induced myokine release influences immune regulation and gut physiology, whereas microbial metabolites, particularly SCFAs, contribute to skeletal muscle metabolism through potential effects on insulin sensitivity, mitochondrial function, and inflammatory responses [13,51,52]. Emerging evidence also suggests that regular physical activity may improve intestinal barrier integrity and promote a microbial environment associated with healthier aging [6,51]. However, many of these mechanistic pathways have been demonstrated primarily in experimental models or mixed adult populations, and confirmation in postmenopausal women remains limited.

Resistance exercise is relevant to postmenopausal care because of its effects on musculoskeletal function, but these effects should be distinguished from proposed microbiome mechanisms. In a six-month randomized trial in postmenopausal women, low-load, high-repetition resistance training improved maximal strength and attenuated lumbar spine bone loss, without favorable changes in fat mass or fat-free soft tissue mass [64]. Separately, a one-year randomized aerobic exercise trial in postmenopausal women demonstrated reductions in total and abdominal adiposity [63]. Neither trial assessed the gut microbiome. The broader gut–muscle literature provides mechanistic context [52], but does not establish that these clinical exercise benefits are mediated by microbial changes.

From a cardiovascular perspective, physical activity remains an important component of risk reduction during and after the menopausal transition [3]. Microbial metabolites and intestinal immune signaling provide plausible pathways through which exercise-related microbial changes might influence vascular physiology [48,53]. However, these sources do not establish microbial mediation of exercise-related cardiovascular benefits in postmenopausal women. Longitudinal intervention studies integrating microbiome and metabolomic profiling with cardiovascular outcomes are needed to test this possibility.

Taken together, current evidence supports regular physical activity as a cornerstone of precision lifestyle interventions for postmenopausal women and should be considered alongside other lifestyle behaviors, including sleep and dietary habits, within an integrated lifestyle framework [69]. Combined aerobic and resistance exercise supports metabolic and musculoskeletal health [63,64], but its effects on microbiome-mediated metabolic aging remain a research question. However, future randomized controlled trials should determine the optimal exercise modalities, intensity, and duration required to achieve sustained microbiome-associated health benefits in this population.

### 6.3. Probiotics, Synbiotics and Phytoestrogens

Probiotics, synbiotics, and phytoestrogens represent complementary microbiome-targeted strategies that have attracted growing interest as potential adjuncts for improving metabolic and menopausal health. Their proposed mechanisms include effects on microbial activity and host signaling [65,70], and microbial biotransformation of dietary isoflavones [66]. Their clinical effects, however, may depend on multiple factors, including baseline microbiome composition, intervention duration, specific formulation, and individual host characteristics [38,70].

Multispecies probiotic formulations have been investigated in postmenopausal women with obesity. In a 12-week randomized, double-blind, placebo-controlled trial, Szulińska et al. reported significant between-group differences in changes in circulating LPS, glucose, insulin, and HOMA-IR following supplementation [42]. A subsequent post hoc analysis reported changes in fecal SCFA concentrations [37]; this analysis should not be considered an independent replication of the original trial. These findings concern a specific formulation and selected surrogate outcomes, and do not establish long-term cardiovascular benefit or justify extrapolation to all probiotic products.

Psychobiotics represent a specialized subgroup of probiotics that target the gut–brain axis. Experimental and clinical studies have suggested potential effects on stress, anxiety, sleep quality, and cognitive function through mechanisms involving microbial metabolites, neurotransmitter signaling, and modulation of the hypothalamic–pituitary–adrenal axis [55]. However, evidence specifically in postmenopausal women remains limited, and current findings should therefore be considered preliminary pending confirmation in larger randomized clinical trials.

Synbiotics are defined as mixtures of live microorganisms and substrates selectively utilized by host microorganisms that confer a health benefit on the host [65]. This consensus definition does not establish metabolic efficacy in postmenopausal women or superiority over probiotics or prebiotics alone. Such benefits require formulation- and population-specific trials. Future studies integrating metagenomic and metabolomic analyses may help determine whether synbiotic interventions can be tailored to individual microbial profiles.

Phytoestrogens, including isoflavones, lignans, and coumestans, illustrate the close interaction between nutrition, the gut microbiome, and host endocrine physiology. Their biological activity depends not only on estrogen receptor interactions but also on microbial biotransformation, particularly the conversion of daidzein into equol, a metabolite with distinct estrogenic activity [66]. Because equol production is restricted to individuals harboring specific gut microbial communities, considerable interindividual variability exists in the response to isoflavone exposure [66,71]. Equol-producing individuals may derive greater benefits from isoflavone interventions for selected menopausal outcomes, although the magnitude and clinical relevance of these differences remain incompletely established [71]. This represents an important example of microbiome-dependent variability in nutritional response and supports further investigation of microbiome-informed nutritional strategies.

Collectively, probiotics, synbiotics, and phytoestrogens illustrate how modulation of the gut microbiome may complement conventional approaches for managing menopausal symptoms and cardiometabolic health. However, substantial heterogeneity in study design, intervention protocols, microbial strains, doses, and microbiome assessment currently limits direct comparisons across studies [38]. Future randomized trials incorporating metagenomic, metabolomic, and clinical phenotyping are needed to identify the patients most likely to benefit from these microbiome-targeted interventions and to determine whether such approaches can meaningfully contribute to precision nutrition in postmenopausal care.

### 6.4. Menopausal Hormone Therapy–Microbiome Interactions

Menopausal hormone therapy (MHT) is the most effective treatment for menopausal vasomotor symptoms and is effective in preventing menopausal bone loss [28,29]. Beyond its established endocrine effects, growing evidence suggests that hormone therapy may interact with the gut microbiome, highlighting a potentially important pharmacomicrobiomic dimension of postmenopausal care [12]. However, the clinical significance of these interactions remains incompletely defined, and current evidence does not yet support the use of microbiome characteristics to guide hormone therapy selection or dosing.

The relationship between MHT and the gut microbiome is particularly relevant to oral estrogen therapy. Oral estrogens undergo hepatic first-pass metabolism and enterohepatic circulation [28,29], creating a theoretical pathway through which microbial β-glucuronidase activity could influence the deconjugation and recirculation of estrogen metabolites [10,11]. Differences in estrobolome activity may therefore contribute to interindividual variation in estrogen metabolism. Nevertheless, direct human evidence demonstrating that variation in estrobolome activity materially alters MHT pharmacokinetics, clinical efficacy, or adverse outcomes remains limited. Transdermal estrogen bypasses hepatic first-pass metabolism and differs pharmacokinetically from oral estrogen [28,29]; however, this should not be interpreted as evidence that transdermal therapy is completely independent of the gut microbiome.

Importantly, human evidence demonstrating menopause-associated alterations in the estrobolome does not establish an effect of MHT on this pathway. Large-scale metagenomic analyses have identified differences in gut microbial composition and β-glucuronidase-related functional capacity between premenopausal and postmenopausal women [7]. However, women receiving hormone therapy were excluded from the relevant analysis [7], and therefore these findings cannot be interpreted as evidence that MHT modifies estrobolome activity. The potential effect of hormone therapy on microbial estrogen metabolism remains an important question for future investigation.

MHT may also influence the gut microbiome indirectly through restoration of estrogen signaling. Estrogen receptors are expressed in intestinal epithelial and immune cells, and estrogen signaling has been implicated in the maintenance of epithelial barrier integrity, mucosal immune regulation, and host–microbiome interactions [12]. Whether restoring estrogen signaling with MHT consistently changes microbial composition or function in women remains uncertain, particularly given the influence of age, diet, body composition, lifestyle, and medication use. Consequently, it remains unclear whether MHT-induced microbiome changes contribute substantially to the established metabolic effects of hormone therapy or instead represent a parallel consequence of restored estrogen signaling.

The progestogen component of combined MHT introduces additional complexity. Different progestogens have distinct pharmacological and metabolic properties [28,29], but evidence regarding their specific effects on the gut microbiome remains sparse. Although micronized progesterone has been proposed as potentially metabolically favorable compared with some synthetic progestogens [67], there is currently insufficient evidence to conclude that differences in microbiome modulation explain these clinical effects or to support microbiome-based selection of progestogen formulations.

Taken together, current evidence supports a potential bidirectional relationship between hormone therapy and the gut microbiome, but the clinical relevance of this interaction remains an emerging research question rather than an established therapeutic principle [11,12]. Future prospective studies combining hormonal measurements, shotgun metagenomics, estrogen metabolomics, microbial functional profiling, and standardized MHT exposure will be necessary to determine whether microbiome characteristics can predict hormone exposure, treatment response, or adverse effects and whether such information can ultimately contribute to personalized menopausal care.

## 7. Clinical Translation and Future Perspectives

The mechanistic framework presented throughout this review, linking estrogen decline, estrobolome remodeling, altered microbial metabolism, and microbiome-mediated metabolic aging, naturally leads to an important clinical question: can these biological insights be translated into individualized strategies for improving postmenopausal health? If the gut microbiome contributes to individual differences in metabolic aging after menopause, characterizing its composition and functional capacity may eventually support more personalized prevention and treatment strategies. This concept lies at the intersection of precision medicine, precision nutrition, and microbiome science. Technologies such as metagenomics, metabolomics, and machine learning are expanding our ability to characterize host–microbiome interactions [68,72]; however, their systematic integration into large, well-characterized postmenopausal cohorts remains limited, delaying translation into routine clinical practice.

### 7.1. Metagenomics, Metabolomics and the Multi-Omics Landscape

Shotgun metagenomics, the untargeted sequencing of microbial DNA within a biological sample, provides a functional characterization of the gut microbiome that extends beyond the taxonomic profiling achievable with 16S rRNA gene sequencing. While 16S sequencing primarily identifies microbial community composition, metagenomics enables characterization of microbial genes and pathways, including those potentially involved in β-glucuronidase activity and other functions relevant to estrobolome biology [7,25]. Applied to postmenopausal cohorts, metagenomic profiling has begun to identify microbial communities that differ not only in taxonomic composition but also in functional capacity, including estrogen-related microbial genes and other metabolic pathways [7]. These functional differences may contribute to interindividual variability in metabolic phenotypes, although prospective validation remains limited.

Metabolomics provides a complementary downstream assessment of microbial and host metabolic activity by measuring small-molecule metabolites in biological samples [68]. In the study of 44 women with menopausal symptoms, fecal metabolomic differences between groups defined by follicle-stimulating hormone concentration involved amino acid and lipid pathways [34]. These findings do not establish validated metabolite signatures of insulin resistance or cardiovascular risk in postmenopausal women. Integrating microbial functional profiling with metabolomics may help connect microbial potential with measured metabolic phenotypes, but prospective validation remains necessary.

Beyond metagenomics and metabolomics, transcriptomics, proteomics, and lipidomics may provide additional information regarding host responses and microbial function. Together, these approaches form a multi-omics framework capable of capturing the complexity of host–microbiome interactions [68,72]. Nevertheless, their application in postmenopausal research remains limited by cross-sectional study designs, relatively small and heterogeneous cohorts, differences in menopausal staging, and limited methodological standardization [8,9]. Consequently, the potential of multi-omics approaches for identifying clinically useful biomarkers and therapeutic targets of microbiome-mediated metabolic aging remains to be established.

From a clinical perspective, multi-omics technologies may eventually allow assessment of microbial function rather than microbiome composition alone [6,68]. Integrating metagenomic, metabolomic, and complementary omics data could facilitate identification of women at increased risk of adverse metabolic trajectories and support the development of precision nutrition and precision medicine strategies. However, standardized analytical pipelines, longitudinal validation, and cost-effectiveness studies are required before these technologies can be incorporated into routine postmenopausal care.

### 7.2. Predictive Microbiome Signatures and Risk Stratification

The clinical value of multi-omics technologies ultimately depends on their ability to generate reproducible biomarkers that support individualized risk assessment and therapeutic decision-making. In postmenopausal women, microbiome-derived signatures represent promising candidates for identifying individuals at increased risk of cardiometabolic and other age-related complications. However, most candidate biomarkers remain at the discovery or early validation stage [6,8,68].

The estrobolome represents a particularly interesting functional candidate because microbial genes and enzymatic activities associated with β-glucuronidase may provide information about the capacity for estrogen deconjugation and enterohepatic estrogen recycling [11,25]. Estrobolome profiling has therefore been proposed as a potential marker of interindividual variability in estrogen metabolism, with possible relevance to endogenous estrogen exposure and hormone therapy. However, this remains a hypothesis rather than a clinically validated biomarker application. Prospective studies directly evaluating whether estrobolome profiling can predict HRT exposure, efficacy, adverse effects, or dose requirements are currently lacking.

Importantly, the available human evidence should not be overstated. Large metagenomic data from the Hispanic Community Health Study/Study of Latinos have demonstrated menopause-associated alterations in microbial composition and estrogen-related functional capacity, including lower microbial β-glucuronidase gene abundance in postmenopausal compared with premenopausal women [7]. However, these findings do not establish that estrobolome characteristics predict HRT response. In particular, the study was designed to characterize microbiome and hormone-related differences according to menopausal status, rather than to determine whether HRT modifies the estrobolome. Therefore, the potential use of estrobolome profiling for HRT personalization remains an important research question rather than an established clinical application.

Among microbiome-associated metabolites, TMAO has been investigated as a cardiovascular risk-associated biomarker. Elevated circulating TMAO concentrations have been associated with increased cardiovascular risk in prospective studies [16]. Nevertheless, whether TMAO assessment improves cardiovascular risk prediction beyond established clinical risk factors, specifically in postmenopausal women, remains to be determined.

The gut–brain axis also offers potential opportunities for microbiome-based risk stratification. Candidate biomarkers, including microbial metabolites and metabolic characteristics of gut microbial communities, have been associated with neuroinflammation, cognitive impairment, and mood-related phenotypes [55,56]. However, current evidence remains insufficient to support routine clinical implementation, particularly in postmenopausal populations.

Taken together, predictive microbiome signatures represent a promising component of future precision medicine, precision nutrition, and pharmacomicrobiomics. However, large prospective longitudinal studies are required to validate candidate biomarkers across diverse populations, establish reproducible analytical thresholds, and determine whether microbiome-informed risk stratification improves outcomes beyond conventional clinical assessment [6,68,72].

### 7.3. Machine Learning and Clinical Integration

The increasing complexity of microbiome multi-omics datasets requires analytical approaches capable of capturing high-dimensional and potentially nonlinear biological interactions. Machine learning has therefore emerged as a promising tool for integrating microbial taxa, functional genes, metabolites, and clinical variables. Applications in gut microbiome research have demonstrated the potential of machine-learning approaches to identify molecular signatures, integrate multi-omics datasets, and generate predictive models of human phenotypes [72]. However, improved model performance in discovery datasets does not necessarily demonstrate clinical utility or generalizability.

The principal challenge is therefore clinical validation rather than algorithm development alone. Many microbiome-based models are developed using relatively small, heterogeneous datasets, while microbiome composition is strongly influenced by factors such as age, diet, medication exposure, geography, and other environmental variables [6]. These sources of variation can contribute to model overfitting and limit transferability across populations [72]. External validation in large, well-characterized postmenopausal cohorts will therefore be essential before microbiome-based predictive models can be considered for clinical use.

Successful clinical integration will also require methodological standardization. Harmonized protocols for microbiome sampling, DNA extraction, sequencing, bioinformatic processing, and metabolomic analysis are necessary to improve reproducibility and comparability across studies [6,72]. Reference datasets incorporating menopausal stage, diet, medication exposure, and relevant demographic characteristics may further improve interpretation of individual microbial profiles.

Ultimately, the clinical value of machine learning will depend not only on predictive accuracy but also on whether its use improves patient outcomes [72]. To date, microbiome-based machine-learning approaches have not been sufficiently validated to establish clinical benefit specifically in postmenopausal populations. Until such evidence becomes available, machine learning should be regarded as a promising translational research tool rather than a validated component of routine postmenopausal care.

### 7.4. Methodological Limitations and Current Gaps

Despite the growing body of evidence linking the gut microbiome to postmenopausal metabolic health, several methodological limitations must be considered. Most human studies are cross-sectional, making it difficult to determine whether microbial alterations contribute to metabolic deterioration or arise as a consequence of menopause-associated metabolic changes [7,9]. Well-designed longitudinal cohorts and interventional studies are therefore needed to establish temporal and mechanistic relationships. In addition, sample sizes remain modest for many multi-omics studies, and geographically concentrated study populations limit generalizability to populations with different dietary, genetic, and environmental characteristics [9].

Although experimental models have provided important mechanistic insights into the gut microbiome–estrobolome axis, clinical evidence remains comparatively limited. Trials evaluating probiotics, prebiotics, dietary interventions, and other microbiome-targeted strategies in postmenopausal women are often characterized by relatively short intervention periods, heterogeneous protocols, and inconsistent clinical endpoints [37,38]. Furthermore, relatively few studies simultaneously assess microbiome composition, microbial function, metabolomics, hormone concentrations, HRT exposure, and long-term cardiometabolic outcomes [8,38]. This limits the ability to determine the relative contribution of microbiome-dependent mechanisms to clinical outcomes.

Additional challenges include the lack of standardized methodologies for microbiome sampling, sequencing, bioinformatic processing, and functional interpretation [6,9]. Differences in menopausal staging, HRT use, dietary habits, medication exposure, and lifestyle factors represent important sources of biological variability that should be consistently addressed in future studies [8,9]. These methodological issues are particularly relevant when attempting to compare microbiome profiles across cohorts or develop clinically applicable predictive signatures.

Despite these limitations, the evidence synthesized in this review supports a plausible and biologically coherent framework linking estrogen decline, estrobolome remodeling, altered microbial metabolism, and microbiome-mediated metabolic aging. However, the extent to which these pathways contribute causally to clinical outcomes remains incompletely established. Bridging the gap between biological plausibility and clinical application will require large prospective studies integrating metagenomics, metabolomics, microbial functional profiling, microbiome-targeted interventions, HRT exposure, and long-term clinical outcomes. Such studies will be essential to determine whether precision nutrition, pharmacomicrobiomics, and microbiome-informed precision medicine can meaningfully improve healthy aging trajectories in postmenopausal women.

## 8. Conclusions

Menopause is a complex biological transition accompanied by substantial endocrine, metabolic, immune, and physiological changes. Increasing evidence indicates that the gut microbiome participates in this process through interactions with estrogen metabolism, host immunity, intestinal barrier function, and microbial metabolite production. The evidence synthesized in this review supports a biologically plausible framework of microbiome-mediated metabolic aging, in which estrogen decline, estrobolome remodeling, altered microbial metabolism, and host responses may collectively contribute to metabolic and systemic changes after menopause. However, the extent to which these microbiome-dependent mechanisms causally contribute to clinical outcomes remains incompletely established.

Dietary modification, fermentable fiber, physical activity, probiotics, synbiotics, phytoestrogens, and clinically indicated hormone therapy may interact with the microbiome through different pathways. Their clinical roles and degree of validation differ, and microbial mediation of benefits remains unproven. Nevertheless, current evidence remains heterogeneous, and microbiome characteristics should not yet be considered established biomarkers for selecting or dosing menopausal therapies.

Future research should prioritize large longitudinal cohorts and well-designed randomized trials integrating shotgun metagenomics, metabolomics, microbial functional profiling, hormone exposure, and clinically relevant outcomes. External validation of predictive models and standardized microbiome methodologies will be essential to determine whether microbiome-derived signatures can provide clinically meaningful risk stratification or guide individualized interventions.

Ultimately, the value of the gut microbiome in menopause may lie less in identifying a single therapeutic target than in providing a framework for understanding the interaction between estrogen biology, metabolism, immunity, and host physiology. Establishing whether this framework can translate into effective precision nutrition, pharmacomicrobiomics, or microbiome-informed interventions remains an important direction for future research.

## Figures and Tables

**Figure 1:**
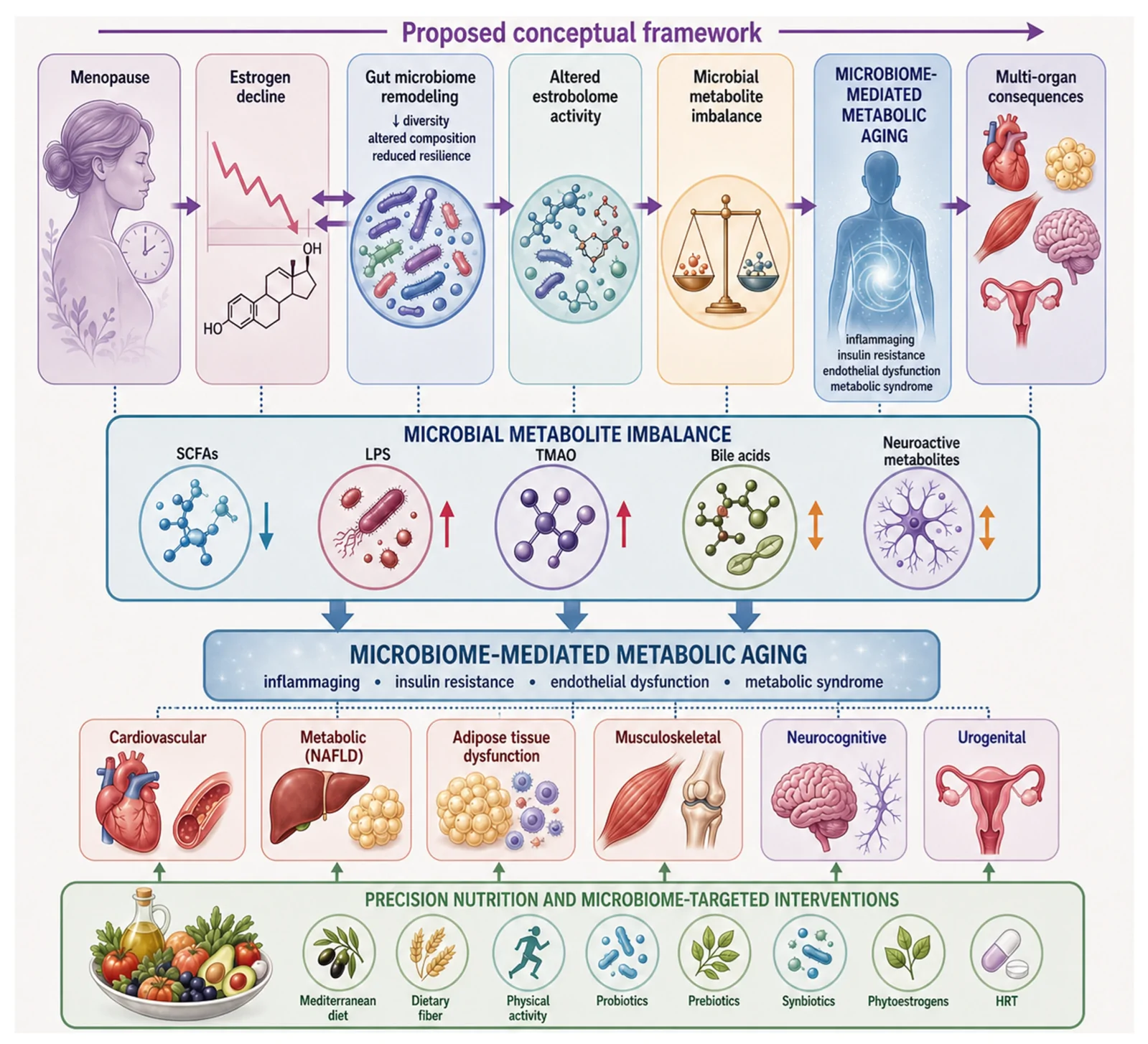
Proposed conceptual framework linking menopause, the estrobolome, gut microbiome remodeling, and microbiome-mediated metabolic aging. The menopausal decline in ovarian estrogen has been associated with changes in gut microbial composition and function, although no consistent menopause-specific diversity pattern has been established [7,9]. These alterations may modify estrobolome activity and estrogen metabolism and contribute to changes in microbial metabolites, including SCFAs, LPS, TMAO, bile acids, and neuroactive metabolites [10,13,14,19]. Collectively, these pathways may contribute to microbiome-mediated metabolic aging, characterized by inflammation, insulin resistance, endothelial dysfunction, and metabolic syndrome, with potential consequences across cardiovascular, metabolic, musculoskeletal, neurocognitive, and urogenital systems [3,6]. Because the gut microbiome is responsive to diet, physical activity, and other environmental exposures, microbiome-targeted interventions represent a potential strategy for supporting metabolic health during and after the menopausal transition.

**Figure 2:**
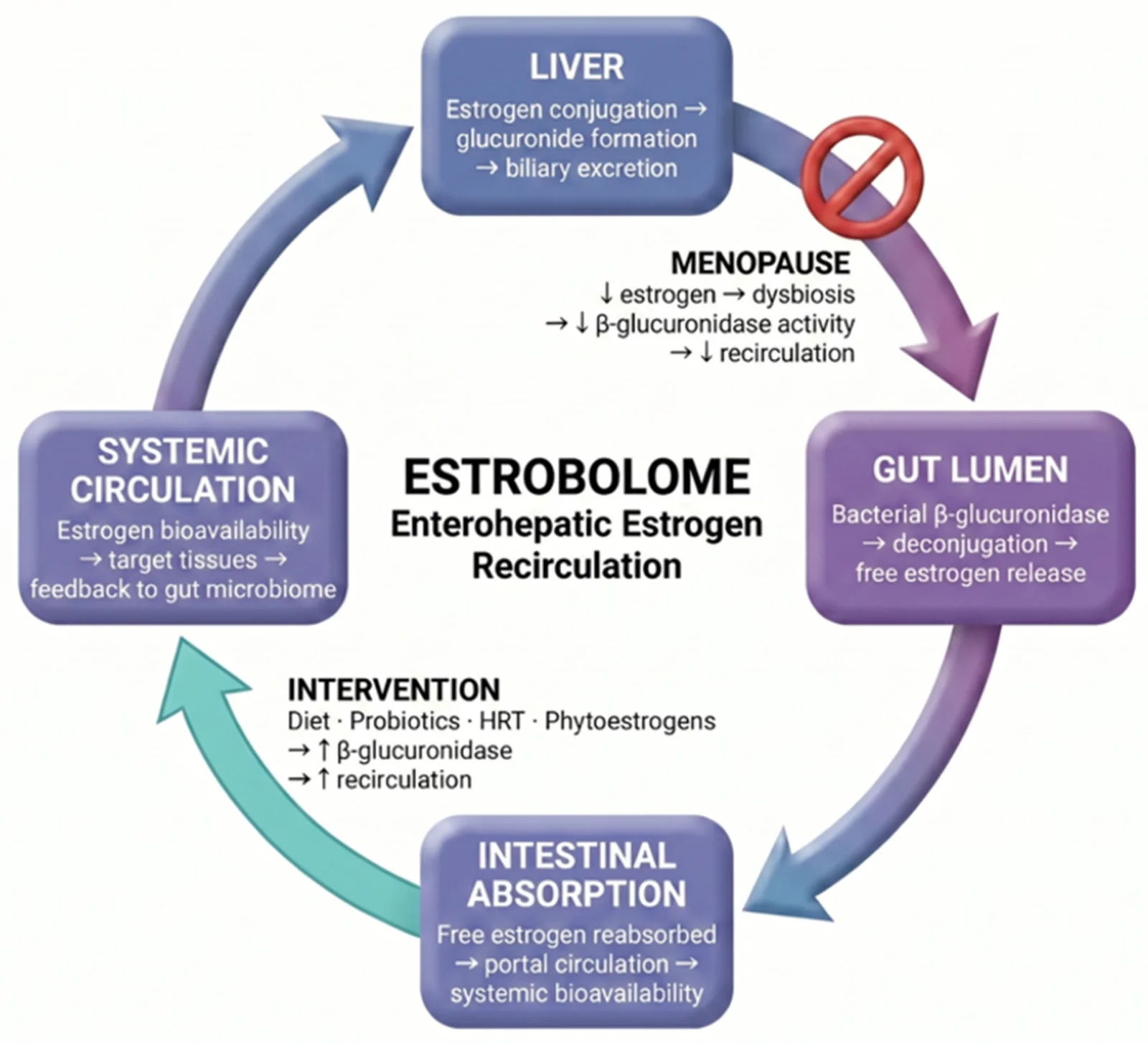
The estrobolome and enterohepatic estrogen recirculation during menopause: proposed mechanistic model. Under physiological conditions, estrogens undergo hepatic conjugation and are excreted into the intestinal lumen via bile [10,11]. Gut microbial β-glucuronidase activity contributes to the deconjugation of estrogen metabolites, allowing unconjugated estrogens to be reabsorbed and returned to the systemic circulation through enterohepatic recirculation [25]. Menopause-associated gut microbiome remodeling may alter estrobolome function and potentially influence estrogen recirculation and systemic estrogen availability [7,8]. The pathway represents a potential interface between endocrine physiology and microbiome-targeted interventions, including dietary and lifestyle strategies. Although the enterohepatic pathway itself is established, the menopause- and intervention-related changes in β-glucuronidase activity and estrogen recirculation illustrated here remain proposed, context-dependent effects and should not be interpreted as consistent responses in postmenopausal women. Microbial gene abundance does not directly measure enzyme activity or enterohepatic estrogen flux, and microbiome-mediated clinical benefits of the listed interventions remain unproven.

**Figure 3:**
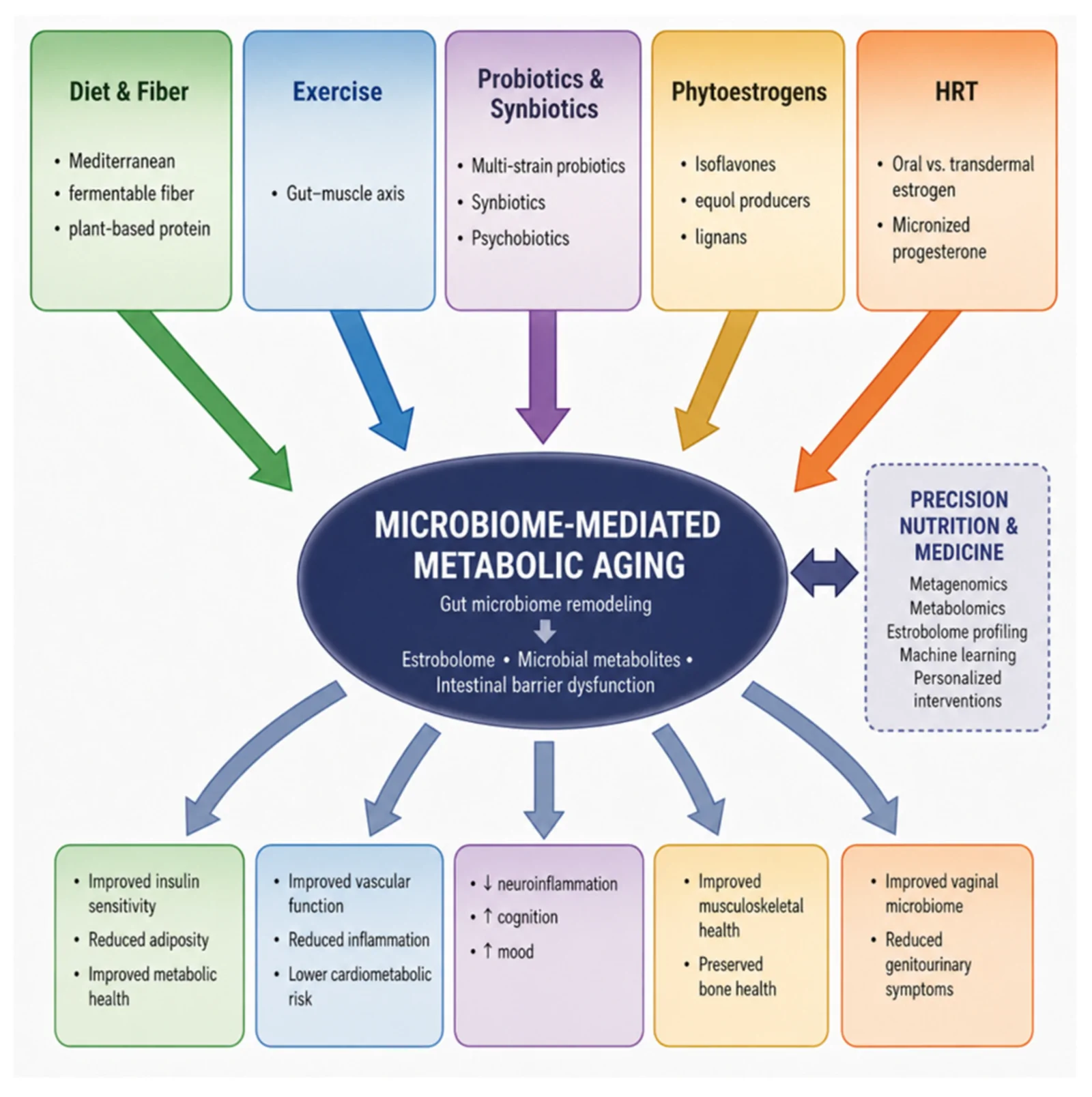
Microbiome-targeted interventions for modifying microbiome-mediated metabolic aging in postmenopausal women.

**Table 1:** Interventions relevant to postmenopausal metabolic health and the microbiome: representative evidence and limitations.

Intervention	Representative Evidence and Population	Findings Relevant to the Review	Interpretation for Postmenopausal Care
Mediterranean diet	NU-AGE dietary intervention in older adults of both sexes [61]; PREDIMED cardiovascular prevention trial [62]	NU-AGE reported diet-associated microbial changes. PREDIMED demonstrated cardiovascular benefit in adults at high cardiovascular risk.	Clinical and microbiome findings come from different studies; microbiome mediation of cardiovascular benefit is not established.
Fermentable fiber	Randomized crossover trials in adults with mild constipation and adults with type 2 diabetes [40,41]	Inulin-type fructans increased bifidobacterial abundance; fecal SCFA responses differed between studies.	Evidence is substrate-specific and not specific to postmenopausal women; microbial changes do not establish clinical benefit.
Plant-rich dietary patterns and protein sources	Human dietary challenge and observational comparisons, alongside animal experiments [44]	TMAO responses to L-carnitine differed between omnivores and vegans/vegetarians.	Supports a diet–microbial metabolism interaction, not proof that plant protein prevents cardiovascular disease through TMAO reduction in postmenopausal women.
Aerobic exercise	One-year randomized trial in sedentary postmenopausal women [63]	Reduced total and abdominal adiposity.	Supports exercise for adiposity-related outcomes; microbiome involvement was not assessed.
Resistance training	Six-month randomized trial of low-load, high-repetition training in postmenopausal women [64]	Improved maximal strength and attenuated lumbar spine bone loss; no favorable effect on fat mass or fat-free soft tissue mass.	Supports specific musculoskeletal outcomes; this trial did not assess microbiome changes or establish microbiome-mediated benefits.
Multispecies probiotics	Randomized trial in postmenopausal women with obesity [42] and subsequent post hoc analysis [37]	Changes in selected metabolic and LPS outcomes; the post hoc analysis reported changes in fecal SCFAs.	Formulation-specific findings on surrogate outcomes; the two reports are not independent replications, and cardiovascular event reduction was not established.
Synbiotics	ISAPP consensus statement on definition and scope [65]	Defines combinations of live microorganisms and selectively utilized substrates that confer a health benefit.	Cited for conceptual context, not as evidence of metabolic efficacy in postmenopausal women; benefits require formulation- and population-specific trials.
Phytoestrogens and isoflavones	Review of equol formation and microbial biotransformation [66]	Conversion of daidzein to equol depends on microbial metabolic capacity.	Provides a mechanistic rationale for variable responses; this source does not establish postmenopausal cardiometabolic efficacy.
Oral MHT	Menopause position statement and clinical guideline [28,29]	Established clinical indications and route-specific considerations; interaction with intestinal estrogen recycling [10,11] is biologically plausible.	Use depends on clinical indications and individual risk assessment; microbiome-mediated benefit is unproven.
Transdermal MHT	Menopause position statement and clinical guideline [28,29]	Avoids hepatic first-pass metabolism; route selection involves clinical risk considerations.	Pharmacokinetic differences do not demonstrate microbiome-mediated benefit or justify microbiome-guided prescribing.
Micronized progesterone	Systematic review of weight, BMI, and glucose metabolism during MHT containing micronized progesterone [67]	Evaluates clinical metabolic outcomes, not microbial mechanisms.	Does not support claims of differential microbiome effects or microbiome-based progestogen selection.

The table presents a narrative comparison of representative sources, not a formal certainty-of-evidence assessment or a treatment recommendation. Study design, population, measured outcomes, and applicability to postmenopausal women are reported explicitly. Clinical effects and microbiome findings are distinguished; neither their coexistence nor biological plausibility establishes microbial mediation. Reviews and consensus documents are retained for mechanistic context, definitions, or clinical guidance and are identified as such. Inclusion does not imply that all listed approaches are recommended as microbiome-targeted therapies.

## Data Availability

No new data were created or analyzed in this study. Data sharing is not applicable to this article.

## Abbreviations

The following abbreviations are used in this manuscript:

16S rRNA	16S ribosomal RNA
BMI	Body mass index
CD14	Cluster of differentiation 14
DNA	Deoxyribonucleic acid
FABP2	Fatty acid-binding protein 2
FFAR2	Free fatty acid receptor 2
FFAR3	Free fatty acid receptor 3
FMO3	Flavin-containing monooxygenase 3
FXR	Farnesoid X receptor
GLP-1	Glucagon-like peptide-1
GPR41	G protein-coupled receptor 41
GPR43	G protein-coupled receptor 43
GSM	Genitourinary syndrome of menopause
HRT	Hormone replacement therapy
IL-1β	Interleukin-1 beta
IL-6	Interleukin-6
LBP	Lipopolysaccharide-binding protein
LPS	Lipopolysaccharide
MASLD	Metabolic dysfunction-associated steatotic liver disease
MD-2	Myeloid differentiation factor 2
MHT	Menopausal hormone therapy
NAFLD	Non-alcoholic fatty liver disease
NF-κB	Nuclear factor kappa B
PYY	Peptide YY
RNA	Ribonucleic acid
SCFAs	Short-chain fatty acids
sCD14	Soluble cluster of differentiation 14
SWAN	Study of Women’s Health Across the Nation
TGR5	Takeda G protein-coupled bile acid receptor 5
TLR4	Toll-like receptor 4
TMA	Trimethylamine
TMAO	Trimethylamine N-oxide
TNF-α	Tumor necrosis factor-alpha
UTI	Urinary tract infection
